# Kinetics of microbial and photochemical degradation of aflatoxin B1 in a sandy loam and clay soil

**DOI:** 10.1038/s41598-022-20727-1

**Published:** 2022-10-07

**Authors:** Julius Albert, Katherine Muñoz

**Affiliations:** iES Landau, Institute for Environmental Sciences, University of Koblenz-Landau, 76829 Landau, Germany

**Keywords:** Natural hazards, Environmental chemistry

## Abstract

In a 28-days experiment, we investigated the dissipation of aflatoxin B1 (AFB1) (0.5–500 ${\upmu }\text {g}\,\text {kg}^{-1}$) by microbial (MD) and photodegradation (PD) in two contrasting soils (sandy loam and clay). Sterile incubation in darkness served as control (C). AFB1 was degraded in all scenarios according to simple first-order kinetics with 50% dissipation times of 20–32 (PD), 19–48 (MD), and 56–65 days (C), respectively. Dissipation rates were significantly lower ($\text {p}<0.001$) in the clay soil than in the sandy loam soil, likely due to photoquenching and strong binding of AFB1 by clay minerals and humic substances. In the sandy loam, dissipation rate of MD decreased in function of initial AFB1 concentration, probably due to toxic effects on degrading microbes. In contrast, in the clay soil the dissipation rate increased with increasing concentration up to 250 $\upmu \text {g}\,\text {kg}^{-1}$, followed by a sharp decrease at 500 $\upmu \text {g}\,\text {kg}^{-1}$, indicating an effect of soil texture on the bioavailability of AFB1 to soil microbes. AFB2a was identified as a transformation product in all scenarios. These results confirm the function of soil for AFB1 degradation, which is modulated by abiotic and biotic processes, soil characteristics and initial AFB1 concentration.

## Introduction

Aflatoxins (AFs) are toxic secondary metabolites produced by several species of the fungal genus *Aspergillus*. The occurrence of AFs in food and feed commodities has been associated with serious health consequences for humans and animals^1^ and substantial economic losses for agriculture^2^ and livestock^3^. Soil is considered a natural habitat for filamentous fungi including aflatoxigenic strains and serves as a reservoir for primary inoculum for the infection of plants^4^. AFs can be synthesized in situ or introduced into the soil when contaminated plant residues or food from storage systems are buried in the soil for natural degradation^5,6^. The presence of AFs in agricultural soils has been reported, with concentrations ranging from $10^{-2}$ to $10^{1}$ $\upmu \text {g}\,\text {kg}^{-1}$^5^. Further, the occurrence of AFs has the potential to alter the ecological balance in soil^6,7^, namely the structure and functions of microbial communities. Specifically, AFs can affect soil bacteria, fungi, and actinomycetes^8^, thus impairing associated soil biogeochemical processes. In the context of assessing the environmental relevance of a toxic pollutant, the question of its persistence in the environment in which it occurs, arises since the rate of dissipation largely determines the duration and intensity of ecotoxicological effects. Dissipation processes in soil are driven by microbial, physical and chemical factors. Since the conditions of the respective degradation processes are different, the rate of dissipation and the resulting transformation products may also vary. The resulting transformation products may be more toxic and persistent than parent compounds^9^, thus investigation on metabolites are essential. Soil has been largely overlooked as a potential sink of AFs and as a matrix in which transformation reactions take place. To understand the environmental relevance of AFs in soil, investigations on the rate at which AFs dissipates from soil and the processes that lead to their dissipation are imperative.

Microbial and enzymatic degradation of AFs has been summarized by Wu et al.^10^ and Verheecke et al.^11^. Most studies have so far focused on the potential application of such approaches for the detoxification of food and feed commodities. Such studies were performed in vitro using bioreactors, liquid and agar cultures, or matrix specific media and carried out with single species or their isolated enzymes which do not originate from the environment in which aflatoxigenic fungi and their toxins normally occur. These include wood decaying fungi^12,13^, microorganisms isolated from soils that are highly polluted with persistent organic pollutants^14,15^, microorganisms that are used in the food processing industry^16^ and microorganisms isolated from the digestive tract^17,18^. Therefore, the reported almost complete degradations of AFB1 within a few hours to days under in vitro conditions may be optimistically high compared to natural degradation in soil. Dissipation of AFB1 in soil was observed at concentrations of $10{-}50\,\text {mg}\,\text {kg}^{-1}$ with nondetectability in less than 6 days^19,20^ and at 10 $\upmu \text {g}\,\text {kg}^{-1}$ where 50% of the initial mass dissipated ($\mathrm{DT}_{50}$) in less than 5 days^5^. After 112 day of incubation, 1.4–14% of the applied AFB1 was mineralized^19,20^. The mineralization rate was about one sixth slower in the silty clay loam as compared to the silt loam soil. Hence, it was concluded that the clay content and organic content of the soil had a negative effect on the degradation and mineralization rate, which was explained by a lower bioavailability due to sorption of aflatoxins in the corresponding soil compartments^20^. AFs are known to have a medium strong sorption affinity for organic carbon^21,22^ and a particularly strong sorption affinity for clay minerals^19,20,23–26^, thus reducing the bioavailability for the degrading microbes^25^. In this context, Goldberg and Angle^26^ have shown that AFB1 adsorption coefficient was about five times higher in a less humic (0.6% $\text {C}_\mathrm{org}$) silty clay loam soil (37.8% clay) compared to a much more humic (2.9% $\text {C}_\mathrm{org}$) silt loam soil (33.6% clay). Further, reduced mineralization of AFB1 in a silt loam soil fortified with $50\,\text {mg}\,\text {kg}^{-1}$ AFB1 compared to the same soil enriched in $10\,\text {mg}\,\text {kg}^{-1}$ AFB1 was observed^20^, indicating an effect of initial AFB1 concentration on the AFB1 degradation rate. Interestingly, the same group^8^ observed that the initial AFB1 concentration was related to the extent of the ecotoxicological effects observed with a continuous decrease of viable population of fungi, bacteria and actinomycetes in a agar media with 1, 100, 10,000 $\upmu \text {g}$ AFB1 $\text {L}^{-1}$. At the highest AFB1 level, the number of viable fungi, bacteria and actinomycetes was reduced by 38–34% compared to the control in the agar media. A similar situation was observed in AFB1 fortified soils where the effects started 2 weeks after AFB1 application and persisted for nearly 6 weeks^8^. When metabolites were identified using thin layer chromatography, the major metabolites detected were AFB2 and to a lesser extent AFG2 and AFG1^19,20^. However, Starr et al.^27^ found only AFB2a as a single transformation product in an aqueous-soil environment product using HPLC-UV and HPLC-MS for analysis. The authors remarked that the use of thin-layer chromatography may have led to misidentification of metabolites.

In AF hot-spot regions, harvest season often coincide with dry periods^4,28^, conditions that are also observed in the soil. As a result of soil dryness, reduced microbial activity and AFs decomposition is likely. Thus, AFs may undergo physicochemical rather than microbial degradation during this season. To date, numerous physical and chemical conditions are known to detoxify aflatoxins in food matrices as summarized by Pankaj et al.^29^ and Guo et al.^30^ including: UV light, organic acids, ammonia, formaldehyde, ozone, sulfites, hydroxides and hypochlorites. These approaches has not been so far investigated in soils, although soils are exposed to UV irradiation in sunny and dry periods. Further, agricultural practices (e.g. fertilization, liming, tillage), plant root exsudation and biochemical transformation reactions can favor the formation of reactive substances in the soil such as organic acids and sulfites, that may initiate chemical degradation of AFs. Another aspect to be considered in degradation process in the soil is the texture and composition, such as clay minerals and humic substances, as these compartments can protect chemicals from degradation reactions due to their steric rearrangement into adsorption sites^31^ or can catalyze physicochemical degradation processes on their surfaces^27,32–35^. So far only two studies investigated the AFB1 degradation under (almost) abiotic conditions. Accinelli et al.^5^ observed no degradation in an autoclaved soil incubated in the dark. Hence, the authors concluded that AFB1 degradation in soil is mainly driven by microbial processes. Starr et al.^27^ observed no AFB1 dissipation in a dry silty loam soil after 60 days of incubation (in dark). Although the soil was not sterilized prior to incubation, microbial activity and thus biodegradation was considered insignificant because of insufficient soil moisture.

Soil is the natural habitat of aflatoxin-producing fungi and a disposal medium for AF contaminated plant residues. However, the processes underlying AFB1 degradation in soil and how these relate to available AFB1 concentration and physicochemical soil properties have not yet been systematically investigated. In addition, only microbial degradation has been studied as a mechanism of aflatoxin decomposition in soil, although aflatoxins in this system are exposed to other reactive abiotic conditions such as sunlight or chemical reagents. Therefore, the aim of this study was to elucidate the dissipation rate of AFB1 in two different soils (sandy loam and clay soil) under abiotic and biotic conditions. For this purpose, soils were amended with 50 $\upmu \text {g}\,\text {kg}^{-1}$ AFB1 and subjected to microbial degradation (MD) and UV light induced photodegradation (PD). Sterile soils amended with 50 $\upmu \text {g}\,\text {kg}^{-1}$ AFB1 and incubated in dark served as control. In addition, it was examined whether increasing initial concentrations of AFB1 (0.5–500 $\upmu \text {g}\,\text {kg}^{-1}$) have an effect on the dissipation rate of AFB1 in soils subjected to MD. The samples were further analyzed for the formation of the previously described metabolites in soil matrices, i.e. AFB2, AFB2a, AFG1 and AFG2. Since clay minerals and humic substances can strongly bind AFs and attenuate light, we assume that (i) AFB1 is less available to soil microorganisms, enzymes and UV light in the more humic and clayey soil resulting in a reduced AFB1 dissipation rate. Because of the potential toxic effect of AFB1 on soil microbes, we expect (ii) a negative relationship between AFB1 dissipation rate and AFB1 fortification level.

## Methods

### Chemicals and reagents

Ultrapure water was used throughout all work (Milli-Q-water purification system, 18.2 M $\Omega \text {cm}^{-1}$, EASYpure II, Millipore Bedford, MA). Acetonitrile (MeCN) and methanol (MeOH) used for extraction, reconstitution, chromatography and preparation of standards were of HPLC grade (Carl Roth, Karlsruhe, Germany). A standard mixture solution with certified concentrations of $20\,\text {mg}\,\text {L}^{-1}$ each for AFB1, AFB2, AFG1, and AFG2 dissolved in MeCN (Sigma-Aldrich, St. Louis, USA) was used for preparation of external calibration standards. A stock solution containing $500\,\text {mg}\,\text {L}^{-1}$ AFB1 was prepared by dissolving 10 mg crystalline AFB1 (from *Aspergillus flavus*, by Sigma-Aldrich, St. Louis, USA) in 20 mL MeCN which was then used for sample fortification. The concentration of the fortification standard was not significantly different from the nominal concentration of $500\,\text {mg}\,\text {L}^{-1}$ (see SI-2 Quality criteria and pretests). A qualitative AFB2a standard was prepared as described by Rushing et al.^36^. Briefly, AFB1 ($2.5\,\text {mg}\,\text {L}^{-1}$) was dissolved in 1 M citric acid solution (Carl Roth, Karlsruhe, Germany) to achieve a nominal concentration of 500 $\upmu \text {g}\,\text {L}^{-1}$. This AFB1 solution was allowed to react for 72 h to form AFB2a. The AFB2a standard was then diluted to 5 $\upmu \text {g}\,\text {L}^{-1}$ with ACN and was used for identification of AFB2a in sample extracts from the degradation experiments. All solutions were stored in the dark at $-20\,^\circ \text {C}$ until analysis.

### Soil characteristics

The degradation experiments were carried out using two soils. The sandy loam soil “R01A” (“RefeSol 01-A”, Fraunhofer IME, Schmallenberg, Germany) and clay soil “L6S” (“LUFA 6S”, LUFA, Speyer, Germany), both served as reference soils from organically managed arable areas (Table 1). Soils were purchased in field-fresh state and conditioned to meet the requirements of OECD 307^37^ (see SI-2 Quality criteria and pre-tests), which was developed to evaluate the rate of transformation of a test substance, and the nature and rates of formation and decline of transformation products. A detailed description of the soil sampling and preparation is found in the supplementary information (see SI-2 Quality criteria and pretests). The soils correspond to the upper soil layer i.e. at 0–20 cm (L6S) and 0–25 cm (R01A) and were homogenized, 2 mm-sieved (stainless steel) and stored at $4\,^\circ \text {C}$ for less than 1 month. These soils were selected to cover a wide range of physicochemical and microbial properties, which are expected to have an influence on the dissipation of AFB1 i.e. organic carbon content, pH, soil texture, microbial biomass and activity (Table 1). The soil organic carbon and clay mineral contents, as reflected in soil texture (clay content), are of particular interest as these soil fractions represent sorption sites for AFs^23,25,38^ as well as may attenuate the UV light^39^. Basal respiration (BR) and glucose-induced respiration (substrate induced respiration, SIR) of the soil were determined using the MicroResp setup^40^ according to Schirmel et al.^41^. BR is the measured soil respiration after addition of water and represents a measure of the respiratory turnover of predominantly native carbon at steady state^42^. Initial soil respiration after addition of a readily available carbon source such as glucose (SIR) is proportional to the mass of metabolically active organisms and therefore serves as a bioindicator of active microbial biomass^43,44^. Total microbial biomass carbon (C_mic_), which includes both the metabolically active and dormant fractions of the soil microbiome was determined using the chloroform fumigation extraction method^45^. Bulk soil was moisture adjusted to 40% water holding capacity and preincubated in dark at $20\,^\circ \text {C}$ for 1 week prior degradation experiments to reestablish equilibrium of microbial metabolism^37^.

**Table 1:** Physicochemical and microbial (mean ± standard deviation, n=3) properties of the tested soils.

Property	R01A	L6S
Soil type	Sandy loam	Clay
Sand (%)	70.5	23.2
Silt (%)	26.1	35.5
Clay (%)	3.4	41.2
$\text {C}_{\mathrm{org}}$ (%)	0.9	1.7
WHC (%)	29.3	42.4
pH ($0.01\,\text {M}\,\text {CaCl}_{2}$)	5.4	7.3
$\text {C}_{\mathrm{mic}}$ ($\text {mg}\,\text {kg}^{-1}$)	95 ± 15	267 ± 8
SIR (mg CO_2_-C $\text {kg}^{-1}\,\text {h}^{-1}$)	3.8 ± 0.9	11.1 ± 2.3
BR (mg CO_2_-C $\text {kg}^{-1}\,\text {h}^{-1}$)	1.8 ± 0.3	3.7 ± 0.6

### Degradation experiments

Microbial degradation experiments were carried out at four fortification levels with 0.5, 5, 50, 250 and 500 $\upmu \text {g}\,\text {kg}^{-1}$ and a blank free of AFB1. Soils were fortified using acid washed quartz sand coated with AFB1 as carrier. Quartz sand was coated with AFB1 using a fortification standard containing $500\,\text {mg}\,\text {L}^{-1}$ AFB1 dissolved in MeCN. MeCN was used instead of MeOH as a carrier solvent for sample fortification to prevent formation of artifactual methoxy aflatoxin species^27^. The solvent was allowed to evaporate for 1h before the fortified sand was added to the soil in order to avoid potential effects of the solvent carrier on soil microorganisms. A sand application rate of 1% was chosen according to the OECD^46^. The blank soil was prepared using the same procedure, but with MeCN. Fortified soil aliquots of 100 g were incubated in 200 mL polypropylene screw cap beakers in triplicate. To maintain aerobic conditions while minimizing water loss through evaporation, a filter was inserted into the screw cap by drilling a 1 cm hole into which polyester filter floss (Symec, JBL, Neuhofen, Germany) was placed.

Photodegradation experiments were carried out with 10 g (dry weight) aliquots of preincubated soils in 70 mL screw cap incubation glass jars. The incubation vessels had a base area of 24.5 $\text {cm}^{2}$ resulting in a uniformly spread soil layer of approximately 3.5 mm thickness. This thickness was sufficient for UV light to penetrate the soil layer. The jars were equipped with a septum for sterile injections and a 2 mm wide vent sealed with two layers of surgical tape (Micropore, 3M, Neuss, Germany) to allow gas exchange while preventing passage of microbial contaminants. Filled vessels were sterilized by autoclaving the soil for 30 min at $121\,^\circ \text {C}$, followed by a second autoclavation run after 2 days in order to prevent potential recolonization by intact spores. Sterility was verified by absence of colony forming units by spreading sterilized soil on surface of sterile agar medium ($15\;g\,\text {L}^{-1}$ agar, $5\;g\,\text {L}^{-1}$ peptone, $2.5\;g\,\text {L}^{-1}$ yeast extract, $1\;g\,\text {L}^{-1}$ glucose, pH 7.0, Carl-Roth, Karlsruhe, Germany). Soils were fortified by injecting 200 µL of of diluted AFB1 fortification solution ($2.5\;g\,\text {L}^{-1}$ in MeCN) using a glass syringe equipped with a sterile filter (PET, 0.2 $\upmu \text {m}$) into the incubation vessels through the septum to obtain a AFB1 soil concentration of 50 $\upmu \text {g}\,\text {kg}^{-1}$. Potential AFB1 extraction losses due to adsorption to the glass material was excluded (see SI-2 Quality criteria and pretests). Soil samples were incubated under UV irradiation from below with a UV fluorescent tube (40W, CLEO Performance N, Philips, Amsterdam, Netherlands). The UV irradiation received by the soil after absorption losses by the glass material had an intensity of $9.1\,\text {W}\,\text {m}^{-2}$ UVA and $0.03\,\text {W}\,\text {m}^{-2}$ UVB. Sterilized and fortified soil incubated in the dark served as control.

Evaporated water (checked gravimetrically) was replenished weekly by sterile injection of ultrapure water. The homogeneous distribution of AFB1 in the fortified soils was evaluated by spike recoveries at day 0 (see SI-2 Quality criteria and pretests, see Table SI-2). All incubation vessels were incubated at $20\,^\circ \text {C}$ and triplicate samples were removed and analyzed at 0, 1, 3, 8, 15, 22 and 28 days after fortification.

### Aflatoxin extraction and analysis

Aflatoxins, namely AFB1, AFB2, AFG1 and AFG2, in the soil samples were extracted with MeCN:H2O (84 : 16, v + v) and analyzed via high performance liquid chromatography with fluorescence detection (HPLC-FLD), according to Albert et al.^38^. AFB2a was analyzed using the same method with excitation and emission wavelength of the fluorescence detector set to 365 and 455 nm. The retention time of AFB2a was determined by injection of the qualitative AFB2a standard (5 $\upmu \text {g}\,\text {L}^{-1}$). All aflatoxins were quantified by external solvent calibration in the range of 0.05–10 $\upmu \text {g}\,\text {L}^{-1}$. During photochemical post-column derivatization, AFB1 is completely converted to AFB2a by conversion of the double bond of the dihydrofuran moiety into hemiacetal derivatives^47^. This allows quantification of AFB2a peaks with the same external solvent calibration as AFB1.

AFB1, AFB2, AFG1, AFG2 and AFB2a were further confirmed using liquid chromatography-high resolution accurate mass spectrometry (LC-HRMS). Retention time and spectra for AFB2a were determined by injection of the qualitative AFB2a standard (5 $\upmu \text {g}\,\text {L}^{-1}$). Target analysis was performed for the [M + H]+ adducts with ionic masses at 313.0715, 315.0860, 329.0650, 331.0800, and 331.0799 m/z for AFB1, AFB2, AFG1,AFG2, and AFB2a respectively. In addition, the corresponding [$\text {M}\,+\,\text {NH}_4$]+ adducts were continuously monitored to confirm the positive findings. The m/z of the [$\text {M}\,+\,\text {NH}_4$]+ adducts were 330.0962, 332.1132, 351.0467, 353.0631, and 353.0624 for AFB1, AFB2, AFG1, AFG2, and AFB2a, respectively. Example chromatograms and spectra can be found in the Supplementary Information (see SI-3 Chromatographic data, Figs. SI-1 and SI-2).

### Data analysis

Data processing and statistical analyses were performed using R (version 4.0.3, R Core Team). Data manipulation, tidying and visualization was done using the the “tidyverse” package (available from https://doi.org/ggddkj)^48^. For all linear models (i.e. calibration, multiple regression and ANOVA models) the assumption of homoscedasticity was checked via scale-location-plots (square root of standardized residuals versus predicted values)^49^ and the normality assumption was assessed via quantile-quantile plots^49^. Outliers were detected using the boxplot method^49^. Extreme points were defined as values above the third quartile + 3x interquartile range or values below the first quartile - 3x interquartile range. Test results were considered as significant when $\text {p}\,<\,0.05$ and as marginally significant (trend of significance) when $\text {p}\,<\,0.1$.

One sample t-test was conducted to evaluate significant differences between the measured AFB1 concentration in the fortification standard and the nominal concentration (see SI-2 Quality criteria and pretests). To check whether the AFB1 concentrations of the glass adsorption test (see SI-2 Quality criteria and pretests) differ between day 0 and day 8, a two sample t-test was performed.

AFB1 dissipation kinetics were assessed by fitting single first order kinetcs (SFO) to data using the Levenberg-Marquardt type fitting algorithm^50^ with the command “nlsLM” (package “minpack.lm”^51^). SFO rate equations were fitted to the AFB1 concentrations changing with incubation time.

1 $$\begin{aligned} c = c_0 \cdot e^{-k_{SFO} \cdot t} \end{aligned}$$

$\text {c}_0$ is the initial AFB1 at time t = 0 (d) and c is the AFB1 concentration at given time t (d) and $\text {k}_\mathrm{SFO}$ ($\text {d}^{-1}$) is the single first order dissipation rate. The resulting regression models were evaluated for their goodness of fit via visual inspection and Efron’s pseudo coefficient of determination ($\text {R}^2$)^52^. According to the OECD307 guideline^37^ SFO kinetics are favored over other kinetic models unless coefficient of determination $\text {R}^2\,<\,0.7$. All models fullfilled these requirements, except for 1 model (L6S, c = 0.5 $\upmu \text {g}\,\text {kg}^{-1},\,\text {MD},\,\text {R}^2$ = 0.593). The insufficient fit of this model was due to an outlier at t = 3. The removal of this outlier before model fitting resulted in a $\text {R}^2$ of 0.765. In addition, there was an outlier in the nonsterile incubated L6S soil contaminated with 250 $\upmu \text {g}\,\text {kg}^{-1}$ on day 3, where the concentration was higher than the corresponding measurement on day 0. This outlier was also removed prior kinetic modeling. AFB1 dissipation kinetics were visualized by plotting normalized AFB1 concentrations $\text {c/c}_0$ against incubation time t which allowed comparison between different incubation conditions and fortification levels. To estimate the rate of AFB1 dissipation under each incubation condition, SFO kinetics were used to determine 50% dissipation times ($\text {DT}_{50}$). These values indicate the time t (d) within which the concentration of the test substance is reduced by 50%.

2 $$\begin{aligned} DT_{50} = \frac{ln 2}{k_{SFO}} \end{aligned}$$

All data used for kinetic modelling can be found in the supplementary information (see SI-1 Raw data for kinetic modelling of AFB1 dissipation, Table SI-1). The processes involved in the dissipation of AFB1 in the soils under the different incubation conditions were investigated with mass balance analysis. The respective fractions, i.e. extractable AFB1, extractable metabolites and non-quantifiable residues, were determined and expressed as a percentage of the initially applied amount of AFB1. The non-quantifiable fraction represents the initially applied amount of AFB1 minus the extractable amount of AFB1 and the metabolite AFB2a. This fraction represents a sum of numerous processes contributing to dissipation such as the formation of bound residues, incorporation of AFB1 carbon into microbial biomass carbon, mineralization, volatilization and transformation (e.g. into other metabolites).

The dissipation kinetics of AFB1 were tested (i) between the different incubation conditions at the same AFB1 fortification level (50 $\upmu \text {g}\,\text {kg}^{-1}$) considering the soil type (see SI-4 Statistical analyses, Tables SI-3, SI-4 and SI-5) and (ii) between the different AFB1 fortification levels at the microbial degradation scenario considering the soil type (see SI-4 Statistical analyses, Tables SI-6, SI-7 and SI-8). The effects of (i) the predictors degradation conditions (“Type”; factor with the three levels “C”, “MD” and “PD”) and soil type (“Soil”; factor with two levels “L6S” and “R01A”) and their interaction was tested using two-way ANOVA model. The effect of (ii) the predictors AFB1 fortification level (“Level”; numeric with the five levels “0.5”, “5”, “50”, “250” and “500”) and soil type (“Soil”; factor with two levels “L6S” and “R01A”) and their interaction on the AFB1 $\text {c/c}_0$ ratio at the end of incubation (day 28) was tested using a multiple regression model. In the case of a significant two-way interaction, post-hoc tests were performed to analyze the effect of the first predictor on the response variable at each level of the second predictor and vice versa. Statistical significance was accepted at the Bonferroni adjusted alpha level.

## Results

### Evaluation of AFB1 dissipation kinetics under the different incubation conditions

**Figure 1:**
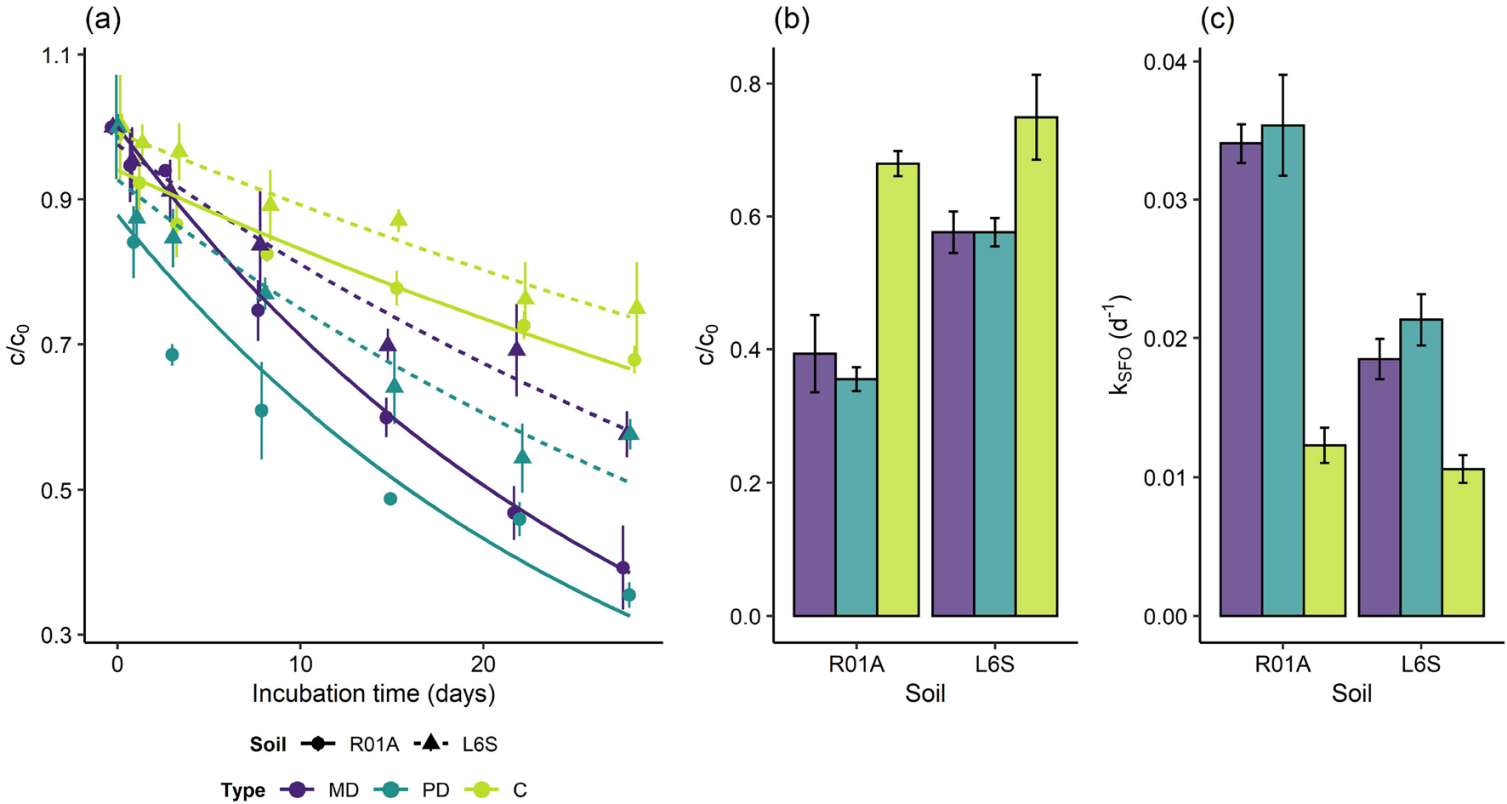
AFB1 dissipation for the sandy loam soil “R01A” (solid lines + points) and clay soil “L6S” (dashed lines + triangles) both fortified with 50 $\upmu \text {g}\,\text {kg}^{-1}$ AFB1 and subjected to microbial degradation “MD” (purple), photodegradation “PD” (dark cyan) and the sterile control in darkness “C” (light green). Curves showing single first order kinetic model fitted to data (**a**), normalized AFB1 concentration $\text {c/c}_0$ at the end of 28-days incubation (**b**) and single first order dissipation rate constants (**c**). Results are presented as mean ± standard deviation (n=3).

The significant decrease in extractable AFB1 concentrations indicates that AFB1 degradation occurred in all investigated soils and incubation conditions (Fig. 1, Table 2). There were significant differences in terms of $\text {c/c}_0$ at the end of the 28-day incubation (Fig. 1a,b) between incubation conditions (F(2,12) = 72.2, $\text {p}\,<\,0.001$). Overall, the order of AFB1 dissipation rate in both soils decreased in the order: PD > MD > C (Fig. 1a,c). The SFO dissipation rate constant in the UV-irradiated soils was slightly faster than microbial degradation by about 3% for the sandy loam and 17% for the clay soil (Fig. 1a,c). Dissipation was significantly lower in the sterile controls than in soils subjected to microbial degradation by about − 65% (sandy loam) and − 39% (clay), and in soils subjected to photodegradation by about − 66% (sandy loam) and − 48% (clay). At the end of the 28-day incubation, the $\text {c/c}_0$ was significantly lower in the sandy loam soil than in the clay soil (F(1,12) = 71.0, $\text {p}\,<\,0.001$, Fig. 1a,b). AFB1 dissipation rate was higher in the sandy loam soil than in the clay soil by about 89%, 67% and 9% for the MD, PD and C, respectively (Fig. 1a,c). Further, a significant interaction between soil and degradation condition (F(2,12) = 5.8, $\text {p}\,=\,0.017$) was found indicating that the dissipation kinetics derived from the degradation conditions was dependent on the soil type or vice versa. In this context, post-hoc analyses (see SI-4 Statistical analyses) had shown that AFB1 dissipated significantly faster in the sandy loam soil than in the clay soil for the MD (F(1,12) = 31.7, $\text {p}\,<\,0.001$) and PD setup (F(1,12) = 46.2, $\text {p}\,<\,0.001$) while the differences between the two soils incubated under C conditions were only marginally significant (F(1,12) = 4.7, $\text {p}\,=\,0.051$).

**Table 2:** Parameters of AFB1 dissipation kinetics for microbial degradation (MD), photodegradation (PD) and the sterile control in darkness (C): AFB1 SFO dissipation rates ($\text {K}_\mathrm{SFO}$) and 50% dissipation times ($\text {DT}_{50}$) and adjusted coefficient of determination ($\text {R}^2$).

Type	Soil	AFB1 concentration level ($\upmu \text {g}\,\text {kg}^{-1}$)	$\text {k}_\mathrm{SFO}$ ($\text {d}^{-1}$)	$\text {R}^2$	$\text {DT}_{50}$ (d)
MD	R01A	0.5	0.034	0.867	20
		5	0.036	0.964	19
		50	0.034	0.977	20
		250	0.033	0.977	21
		500	0.03	0.901	23
	L6S	0.5	0.014	0.772	48
		5	0.015	0.833	48
		50	0.018	0.907	37
		250	0.02	0.933	35
		500	0.016	0.885	43
PD	R01A	50	0.035	0.867	20
	L6S	50	0.021	0.888	32
C	R01A	50	0.012	0.842	56
	L6S	50	0.011	0.861	65

### Effects of initial AFB1 concentration on microbial degradation

**Figure 2:**
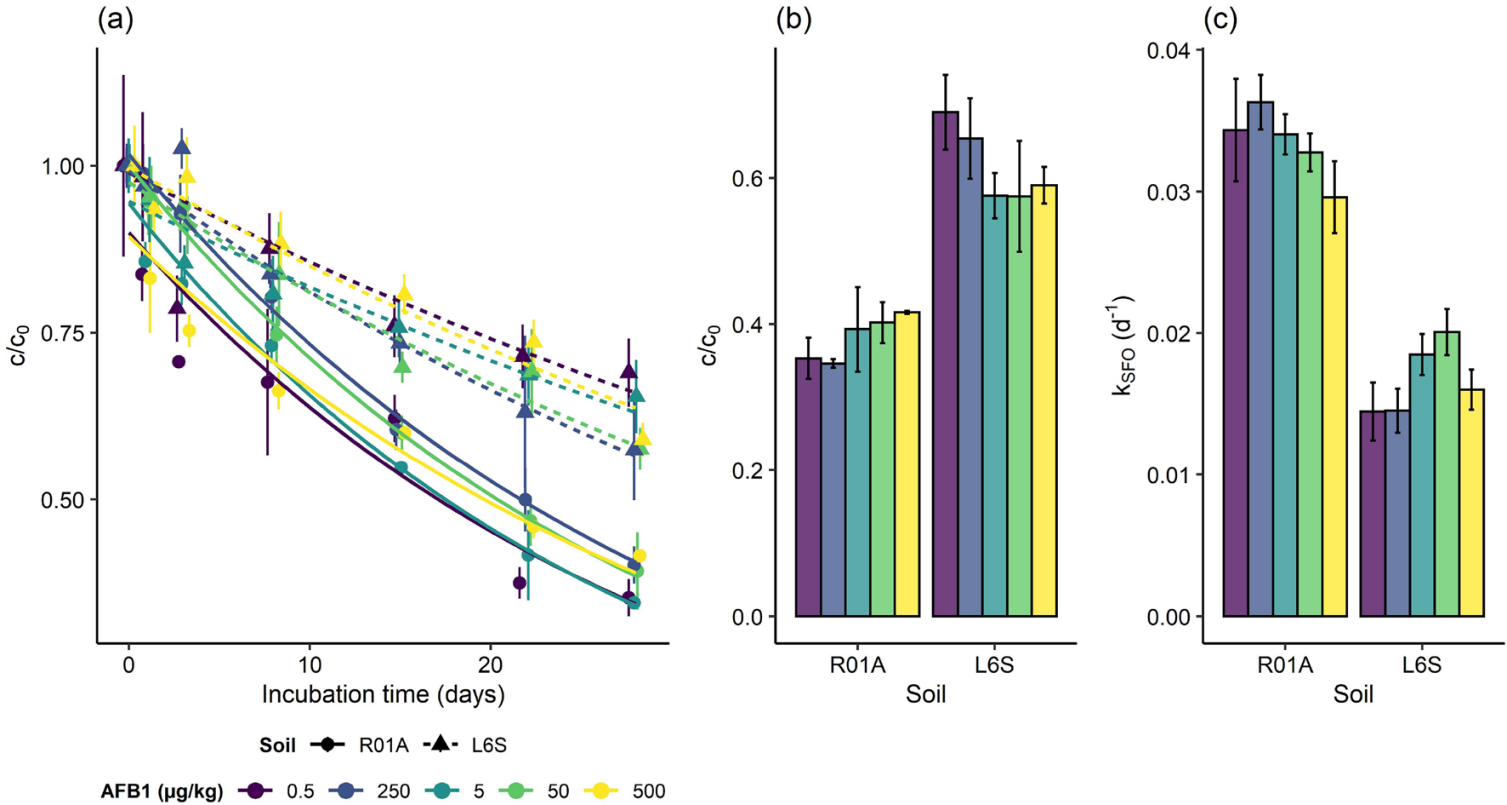
AFB1 dissipation for the sandy loam soil (“R01A”) and clay soil (“L6S”) amended with 0.5, 5, 50, 250 and 500 $\upmu \text {g}\,\text {kg}^{-1}$ AFB1 and incubated under nonsterile (MD) conditions. Curves showing single first order kinetic model fitted to data (**a**), normalized AFB1 concentration $\text {c/c}_0$ at the end of 28-days incubation (**b**) and single first order dissipation rate constants (**c**).

AFB1 dissipated to varying degrees in the two tested nonsterile incubated soils at the different AFB1 fortification levels (Fig. 2, Table 2). The dissipation speed in terms of c/c_0_ at the end of 28-days incubation (Fig. 2a,b) was significantly different between the soils (t(26) = − 12.0, $\text {p}<0.001$) and AFB1 fortification levels (t(26) = −  2.2, $\text {p}=0.040$). Further, the significant interaction between soil type and AFB1 fortification level (t(26) = 2.8, $\text {p}=0.009$) indicates that the concentration dependant AFB1 dissipation was differently affected by the two soil types. Post-hoc analyses (see SI-4 Statistical analyses) showed that, there was a significant positive relationship between the AFB1 fortification level and the $\text {c/c}_0$ ratio for the clay soil (F(1,26) = 4.7, p = 0.04), while a marginally significant negative relationship was observed for the sandy soil (F(1,26) = 3.5, $\text {p}\,=\,0.074$). The negative relationship between AFB1 fortification level and dissipation rate constant was consistent for the whole fortification range in the sandy loam soil (Fig. 2a,c). In contrast, for the clay soils the dissipation rate increased with increasing AFB1 fortification levels from 0.5 to 250 $\upmu \text {g}\,\text {kg}^{-1}$ and then decreased at the highest level (500 $\upmu \text {g}\,\text {kg}^{-1}$) almost to the level of the dissipation rate of the first two levels (0.5–5 $\upmu \text {g}\,\text {kg}^{-1}$).

**Figure 3:**
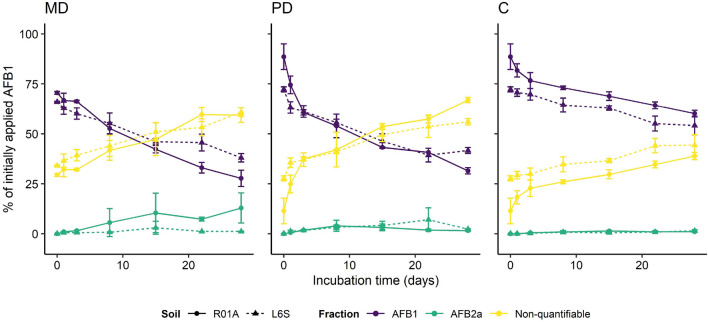
Processes of AFB1 dissipation for the sandy loam soil (“R01A”) and clay soil (“L6S”) incubated (28 days) under dark-abiotic (C), UV irradiated (PD) and nonsterile (MD) conditions. Extractable AFB1 (purple), extractable AFB2a (cyan) and non-quantifiable (yellow) fractions are given as percentage of initially applied AFB1. Results are expressed as mean ± standard deviation (n = 3).

### AFB1 dissipation processes and formation of AFB2a

A constant decrease of the extractable AFB1 fraction and a constant increase of the non-quantifiable fraction was observed for both soils and all incubation conditions during the 28-days incubation (Fig. 3). AFB2a was detected as a transformation product under all degradation conditions, while no AFG2, AFG1 or AFB2 was found (see SI-3 Chromatographic data, Fig. 3). The transformation rate of AFB2a differed between the two soils and the three different degradation conditions. At the end of incubation period 12.9 ± 7.6 (MD), 1.5 ± 0.4 (PD) and 1.0 ± 0.3 (C) % of the initially applied AFB1 was found as AFB2a fraction in the sandy loam soil and 1.1 ± 0.2 (MD), 2.3 ± 0.2 (PD) and 1.4 ± 0.6 (C) in the clay soil. Considerably more AFB2a was found in the MD than in the PD and C samples but the variation was extremely high i.e. 50% of the samples had a coefficient of variation greater than or equal to 33% and 25% of the samples had a coefficient of variation greater than or equal to 52%. In particular, the MD samples showed a very high coefficient of variation up to 128% (R01A, MD, day 8). Only trace concentrations of AFB2a were detected in the sterile control. Throughout the 28 days incubation period, a higher AFB2a formation rate was observed for the MD setup in the sandy loam soil than in the clay soil (Fig. 3). This pattern was not observed for the C and PD conditions, where AFB2a concentrations were nearly equal for both soils, with one exception in the clay soil in PD on day 22, where the AFB2a concentration in the clay soil was 4 times that of the sandy loam soil. However, the relative standard deviation of this time point was 86%. A steady increase in the non-quantifiable fraction was observed over time for all soils and treatments. At the end of incubation 59.4 ± 3.7 (MD), 67.0 ± 1.3 (PD) and 38.9 ± 1.8 (C) of the initially applied AFB1 was found as nonextractable fraction in the sandy loam and 60.9 ± 2.2 (MD), 56.0 ± 1.6 (PD) and 44.4 ± 5.2 (C) in the clay soil. For the MD and PD, this fraction was the most significant at the end of the incubation experiment. The non-quantifiable fraction was nearly the same for both soil for the MD. However, for the PD the non-quantifiable fraction was considerably higher in the sandy loam soil than in the clay soil, while the opposite pattern was observed for the sterile control.

## Discussion

Dissipation of AFB1 and formation of AFB2a occurred in all soils and under all incubation conditions, and the dissipation rate was significantly affected by soil type and degradation scenario. In both soils, the rate of AFB1 degradation in the PD- and MD-treated soils was of the same order of magnitude but was significantly higher than in the controls, as expected. However, the AFB1 dissipation kinetics observed for the PD and MD are much slower than in previous studies. In contrast, a considerable AFB1 dissipation was observed in the abiotic control. This is contrary to the general assumption that aflatoxins are almost recalcitrant to abiotic degradation in soil^6^.

When subjected to microbial degradation, AFB1 dissipated with $\text {DT}_{50}$ values of 19–23 days for the sandy loam soil and 35–48 days for the clay soil. These are much higher than the $\text {DT}_{50}$ value of 4.1 obtained by Accinelli et al.^5^, who used a similar fortification level of 10 $\upmu \text {g}\,\text {kg}^{-1}$. Even at concentrations thousands of times higher ($10{-}50\,\text {mg}\,\text {kg}^{-1}$), AFB1 could no longer be detected in less than 6 days^19,20^. One reason for the discrepancy between the dissipation rate in this study and other studies may be differences in the soil moisture conditions as this greatly affects the physiological state of microorganisms and the functionality of soil enzymes^53^. In former studies, a moisture content of 80-100% field capacity was reported^5,19,20^, other to the 40% in this work. In this regard, a recent study^54^ observed significantly lower microbial degradation of AFB1 in an artificial soil at 30% compared to 50% WHC. It should be noted that AFs degradation in real scenarios may take place under dryer environmental conditions. Therefore, the rapid degradation rates with $\text {DT}_{50}$ of $<\,5$ days reported previously^5,19,20^ may underestimated the persistence of aflatoxins in the soil. Another reason for the discrepancy between the dissipation rate in this study and others  is that reference soils from the European region were used in this study and hence it is unlikely that the microorganisms living in these soils have ever been exposed to AFs. Thus, the enzymes involved in AFB1 degradation may be less effective than the enzymatic repertoire of microbes regularly exposed to aflatoxins.

The dissipation of AFB1 subjected to photolytic degradation was comparable in magnitude to microbial degradation. In the present study, $\text {DT}_{50}$ values of 20 days for the sandy loam soil and 32 days for the clayey soil were observed for the photolytic degradation. These $\text {DT}_{50}$ values are much higher than for photodegradation in other food or liquid matrices which are in the range of few minutes to hours^55^. This discrepancy is attributable to the high light attenuation effect of soil, as a soil layer as thin as 0.5 mm is already sufficient to block about 95% of the incident light^56^. Thus, it is expected that photolytic degradation is mainly limited to AFB1 contaminated material lying on top of the soil and the top layer of the soil. AFs are expected to accumulate mainly in the soil surface layer^26^, thus photodegradation is likely to be of great importance for the degradation of AFs in contaminated soil.

In the sterile controls, a significant dissipation of AFB1 was observed with $\text {DT}_{50}$ of 56 days for the sandy loam soil and 65 days for the clay soil. Furthermore, the presence of AFB2a in the sterile controls suggests that the dissipation of AFB1 observed is at least partly due to chemical degradation. This is contrary to the general assumption that AFs are almost recalcitrant to abiotic degradation in soil^5,6^. However, it is already known that the conversion of AFB1 to AFB2a can occur nonenzymatically in the presence of organic acids^36,57,58^ that are also present in soil matrices^59^. Thus, chemically mediated degradation may be one of the underlying mechanism for the formation of AFB2a in the abiotic controls. In addition, it is possible that the soil enzymes were not deactivated during autoclaving^60^, so that degradation of AFB1 may also have occurred by intact soil enzymes. Contaminated plant material is frequently incorporated into the soil post-harvest in the dry season^4,28^ with limited microbial activity. Soil enzymes often remain active during drought^61^ thus biochemical degradation could play an important role in the decomposition of AFs in the soil.

Regardless of incubation conditions, the degradation rate of AFB1 was significantly slower in the clay soil as compared to the sandy loam soil. These soils differ in physicochemical and microbial properties such as texture, organic carbon content, pH and microbial biomass and activity (Table 1). Although the microbial biomass (C_mic_) and activity (BR, SIR) was around 2–3 times higher in the clay soil as compared to the sandy loam, the microbial dissipation of AFB1 was significantly lower in the clay soil, by about 89% compared to the sandy loam soil. This suggests that soil texture affected the availability of AFB1 for microbial degradation which is consistent with the results of Angle^20^. Medium strong sorption of AFB1 to soil organic carbon has been reported^21,22^. However, in this study, both soils are below 2% organic carbon content, and thus not considered as organic soils in which a higher probability of interaction between aflatoxin B1 and organic carbon would be expected. In addition, soil enzymes can also be sorbed to clay minerals in the soil^62^, restricting their activity. AFB1 is relatively stable in the pH range of the soils studied (5.4 and 7.3) ^63^. However, it was found that the binding strength of AFs^64^ and soil enzymes^65^ to clay minerals decreases significantly with increasing acidity^64,65^. To scrutinize the actual influence of soil pH on the bioavailability to soil microbes and thus on AFB1 biodegradation rate, further studies are needed on other soils at different pH gradients. Soil is known to attenuate light transmission, however the degree of this effect is driven by the soil texture, namely organic carbon and clay minerals. Organic substances such as humic substances and organic ions can act as photoquenchers that delay the photodegradation of a substance^39^. The substance to be degraded and the photoquenching organic ion can be sorbed together on the surfaces of the clay minerals, thus keeping the organic cations and the organic matter at an optimal distance and orientation for the energy transfer processes^66^. The clay mineral itself can also provide photostabilization by charge transfer from the excited organic molecules to Fe^3+^ ions in the crystal structure of the clay mineral^66–68^. However, it remains to be clarified which processes were actually responsible for the reduction in the dissipation rate of photolytic degradation.

It was found that the initial concentration of AFB1 affected the microbial degradation. A significant increase in degradation rate with increasing AFB1 concentration was observed for the clay soil (with a sharp decrease at the highest concentration), while for the sandy loam soil AFB1 concentrations had a marginally significant negative effect on degradation. In this context, Angle^20^ observed a slightly reduced mineralization rate during the first 20 days in a silt loam soil amended with $10\,\text {mg}\,\text {kg}^{-1}$ compared to an amendment of 50 $\upmu \text {g}\,\text {kg}^{-1}$. The same group also observed a negative effect of AFB1 (1, 100, 10,000 $\upmu \text {g}\,\text {kg}^{-1}$) on the population of bacteria, actinomycetes and fungi in an agar medium and in a silt loam soil during the first 28 days after AFB1 application^8^. While these negative effects could be confirmed for the sandy loam soil, the opposite is was observed for the clay soil. This discrepancy may be explained by the interrelationship between sorption/desorption of AFB1 to clay minerals^19,20,23–26^ and humic substances^21,22^ and the effect on the bioavailability. As the desorption/adsorption coefficient of a given substance is a function of the substance concentration, there is consequently a higher fraction of AFB1 dissolved in soil pore water and a lower fraction adsorbed to sorption sites. Thus, the increase in dissipation rate over the first four AFB1 fortification levels (0.5–250 $\upmu \text {g}\,\text {kg}^{-1}$) could be due to the increase in bioavailable concentration. At the highest level, the bioavailable concentration may surpassed the lowest concentration with detrimental effects on the microbial community, resulting in a decline of the dissipation rate. This proposed mechanism cannot be conclusively demonstrated from the present results. A classical ecotoxicity assay for the dose-dependent effects of AFB1 on microbial activity, biomass, and community structure could provide information on the dose-dependent effects on the rate of degradation.

Mass balance analysis showed that a large portion of the dissipated AFB1 was contained in the non-quantifiable fraction for all incubation conditions. However, it is unclear to what extent this non-quantifiable residue is due to volatilization of the parent compound, complete mineralization to $\text {CO}_2$, formation of bound residues, or incorporation of AFB1 carbon into microbial biomass. Volatilization as a cause for the increase in the non-quantifiable fraction seems not plausible, since no aflatoxin is known to be volatile under normal conditions ($20\,^\circ \text {C}$, 1 atm). In previous studies, only minor mineralization of AFB1 was observed in nonsterile soils, namely 14% in 112 days^19^ and 1.4 to 8.1% in 112 days^20^, while $\text {DT}_{50}$ values $<\,5$ days were observed. Significant mineralization therefore remains unlikely compared to the other reasons given previously. Incorporation into the microbial biomass seems unlikely as an exclusive process in light of the fact that a significant non-quantifiable fraction was also detected in the sterile soils (C and PD). Therefore it is likely that the discrepancy between the $\text {DT}_{50}$ determined in the present study and in previous studies was due to a formation of bound residues that could not be removed from the soil matrix by the extraction procedure. The bound residues may not only include the parent compound AFB1, but also the metabolites formed. Hence, it is also possible that the metabolites formed could not be extracted by the extraction procedure used. A classic radiotracer analysis using radiolabelled standards or the application of further extraction steps or more sophisticated analytical methods, which are also able to detect large parts of the non-extractable residues^69^, could provide further information on the fate of AFs in the soil.

## Conclusion

The present study focussed on the degradation and transformation processes contributing to the dissipation of AFB1 in soil, namely microbial degradation and UV light-induced photodegradation. AFB1 dissipated in all soils and incubation conditions and AFB2a was detected as metabolite. The results clearly indicated that the dissipation of AFB1 was significantly affected by the incubation conditions, soil type and initial AFB1 fortification level. The largest fraction of dissipated AFB1 was found in the non-quantifiable fraction indicating that soil-bound residues of the parent compound and/or metabolites were formed. Regardless of the soil tested, a clear pattern emerged in which AFB1 dissipation and AFB2a formation were significantly higher in PD and MD treated soils than in the sterile control. AFB1 dissipation rates for the PD and MD treatments were of a similar magnitude, with the PD treatment being slightly faster. Due to the low penetration depth of UV light in soil, photodegradation is expected to be limited to the uppermost soil layers, so that AFB1 degradation in deeper soil layers is likely to be dominated by microbial degradation. A negative effect of initial concentration on AFB1 dissipation rate was observed for the sandy loam soil but not for the clay soil, which is probably explained by the sorption-induced reduction in bioavailability due to the higher clay mineral content. Although the dissipation rates in the sterile controls were much lower than microbial and photodegradation, biochemical degradation in dark could play an essential role in the degradation of AFB1 when conditions are unfavorable for microbial degradation, such as during extreme drought. Altogether, these results suggest that photolytic and microbial degradation processes are particularly important in the breakdown and deactivation of AFB1 in soil, although these processes depend on the soil properties. The results of this study contribute to a better understanding of the fate and importance of AFs as micropollutants in the environment and illustrate the importance of soil properties for the dissipation processes of AFB1.

## Supplementary Information

Supplementary Information.

## Data Availability

All data generated or analysed during this study are included in this paper and its supplementary information. Additional data related to this paper may be requested from the authors on reasonable request.
