# Supplementary material for: Kinetics of microbial and photochemical degradation of aflatoxin B1 in a sandy loam and clay soil
Source: Sci Rep. 2022 Oct 7;12:16849. doi: 10.1038/s41598-022-20727-1 (PMC9546847; doi:10.1038/s41598-022-20727-1)
Supplement: Supplementary file 1 — Supplementary Information. [file 41598_2022_20727_MOESM1_ESM.pdf]

Kinetics of microbial and photochemical degradation of aflatoxin B1 in a sandy loam and clay soil

Julius Albert[1] **and Katherine Muñoz**[2,*]

[1]iES Landau, Institute for Environmental Sciences, University of Koblenz-Landau, 76829 Landau, Germany, Albert.j@uni-landau.de
[2]iES Landau, Institute for Environmental Sciences, University of Koblenz-Landau, 76829 Landau, Germany, munoz@uni-landau.de
[*]munoz@uni-landau.de

ABSTRACT

In this supplementary information, you will find the raw data as well as additional information on our experiments, chromatography and statistical analyses that support our results.

SI-1 Raw data for kinetic modeling of AFB1 dissipation

Table 1. Calculated AFB1 and AFB2a concentrations in the sterile (PD+C) and nonsterile (MD) soils fortified with 0.5. 5, 50, 250 and 500 μg kg^{-1} AFB1. Values below the detection limit (defined as below the lowest calibration standard i.e. 0.05 μg L^{-1}) are shown as < LOD.

Soil	Fortification level (μg kg^{-1})	Day	Replicate	Degradation Type	AFB1 (μg kg^{-1})	AFB2a (μg kg^{-1})
R01A	0.5	0	1	MD	0.32	-
R01A	0.5	0	2	MD	0.36	-
R01A	0.5	0	3	MD	0.32	-
R01A	0.5	1	1	MD	0.30	-
R01A	0.5	1	2	MD	0.28	-
R01A	0.5	1	3	MD	0.27	-
R01A	0.5	3	1	MD	0.24	-
R01A	0.5	3	2	MD	0.24	-
R01A	0.5	3	3	MD	0.24	-
R01A	0.5	8	1	MD	0.20	-
R01A	0.5	8	2	MD	0.22	-
R01A	0.5	8	3	MD	0.27	-
R01A	0.5	15	1	MD	0.21	-
R01A	0.5	15	2	MD	0.22	-
R01A	0.5	15	3	MD	0.20	-
R01A	0.5	22	1	MD	0.12	-
R01A	0.5	22	2	MD	0.13	-
R01A	0.5	22	3	MD	0.13	-
R01A	0.5	28	1	MD	0.11	-
R01A	0.5	28	2	MD	0.12	-
R01A	0.5	28	3	MD	0.13	-
L6S	0.5	0	1	MD	0.27	-
L6S	0.5	0	2	MD	0.32	-
L6S	0.5	0	3	MD	0.36	-
L6S	0.5	1	1	MD	0.28	-
L6S	0.5	1	2	MD	0.31	-

L6S	0.5	1	3	MD	0.34	-
L6S	0.5	3	1	MD	0.24	-
L6S	0.5	3	2	MD	0.24	-
L6S	0.5	3	3	MD	0.27	-
L6S	0.5	8	1	MD	0.27	-
L6S	0.5	8	2	MD	0.30	-
L6S	0.5	8	3	MD	0.27	-
L6S	0.5	15	1	MD	0.24	-
L6S	0.5	15	2	MD	0.26	-
L6S	0.5	15	3	MD	0.23	-
L6S	0.5	22	1	MD	0.21	-
L6S	0.5	22	2	MD	0.22	-
L6S	0.5	22	3	MD	0.24	-
L6S	0.5	28	1	MD	0.22	-
L6S	0.5	28	2	MD	0.24	-
L6S	0.5	28	3	MD	0.20	-
R01A	5	0	1	MD	4.34	-
R01A	5	0	2	MD	4.50	-
R01A	5	0	3	MD	4.70	-
R01A	5	1	1	MD	3.90	-
R01A	5	1	2	MD	3.72	-
R01A	5	1	3	MD	3.98	-
R01A	5	3	1	MD	3.74	-
R01A	5	3	2	MD	3.85	-
R01A	5	3	3	MD	3.56	-
R01A	5	8	1	MD	3.16	-
R01A	5	8	2	MD	3.37	-
R01A	5	8	3	MD	3.35	-
R01A	5	15	1	MD	2.50	-
R01A	5	15	2	MD	2.44	-
R01A	5	15	3	MD	2.49	-
R01A	5	22	1	MD	1.53	-
R01A	5	22	2	MD	2.03	-
R01A	5	22	3	MD	2.08	-
R01A	5	28	1	MD	1.54	-
R01A	5	28	2	MD	1.55	-
R01A	5	28	3	MD	1.59	-
L6S	5	0	1	MD	3.30	-
L6S	5	0	2	MD	3.32	-
L6S	5	0	3	MD	3.13	-
L6S	5	1	1	MD	3.26	-
L6S	5	1	2	MD	2.85	-
L6S	5	1	3	MD	3.13	-
L6S	5	3	1	MD	2.78	-
L6S	5	3	2	MD	2.86	-
L6S	5	3	3	MD	2.69	-
L6S	5	8	1	MD	2.42	-
L6S	5	8	2	MD	2.68	-
L6S	5	8	3	MD	2.78	-
L6S	5	15	1	MD	2.32	-
L6S	5	15	2	MD	2.61	-
L6S	5	15	3	MD	2.44	-
L6S	5	22	1	MD	2.20	-
L6S	5	22	2	MD	2.39	-
L6S	5	22	3	MD	2.10	-

L6S	5	28	1	MD	2.27	-
L6S	5	28	2	MD	1.93	-
L6S	5	28	3	MD	2.17	-
R01A	50	0	1	MD	35.09	0.00
R01A	50	0	2	MD	35.05	0.00
R01A	50	0	3	MD	35.72	0.00
R01A	50	1	1	MD	34.29	0.51
R01A	50	1	2	MD	34.57	0.59
R01A	50	1	3	MD	31.37	0.46
R01A	50	3	1	MD	33.06	0.86
R01A	50	3	2	MD	33.49	0.82
R01A	50	3	3	MD	32.91	0.80
R01A	50	8	1	MD	24.95	7.24
R01A	50	8	2	MD	27.90	0.85
R01A	50	8	3	MD	26.19	0.77
R01A	50	15	1	MD	22.19	0.78
R01A	50	15	2	MD	20.91	4.63
R01A	50	15	3	MD	20.35	11.11
R01A	50	22	1	MD	16.90	4.29
R01A	50	22	2	MD	17.59	4.05
R01A	50	22	3	MD	15.06	3.30
R01A	50	28	1	MD	14.59	4.46
R01A	50	28	2	MD	15.44	4.54
R01A	50	28	3	MD	11.56	11.42
L6S	50	0	1	MD	33.10	0.00
L6S	50	0	2	MD	33.07	0.00
L6S	50	0	3	MD	32.78	0.00
L6S	50	1	1	MD	30.22	0.19
L6S	50	1	2	MD	30.93	0.22
L6S	50	1	3	MD	33.18	0.42
L6S	50	3	1	MD	29.97	0.27
L6S	50	3	2	MD	31.53	0.30
L6S	50	3	3	MD	28.69	0.32
L6S	50	8	1	MD	29.80	0.50
L6S	50	8	2	MD	24.77	0.33
L6S	50	8	3	MD	28.28	0.43
L6S	50	15	1	MD	22.82	0.60
L6S	50	15	2	MD	22.38	0.58
L6S	50	15	3	MD	23.88	3.54
L6S	50	22	1	MD	21.72	0.58
L6S	50	22	2	MD	25.23	0.62
L6S	50	22	3	MD	21.49	0.57
L6S	50	28	1	MD	19.51	0.69
L6S	50	28	2	MD	17.81	0.52
L6S	50	28	3	MD	19.69	0.57
R01A	250	0	1	MD	169.54	-
R01A	250	0	2	MD	158.86	-
R01A	250	0	3	MD	166.07	-
R01A	250	1	1	MD	159.52	-
R01A	250	1	2	MD	158.09	-
R01A	250	1	3	MD	171.29	-
R01A	250	3	1	MD	151.96	-
R01A	250	3	2	MD	163.04	-
R01A	250	3	3	MD	143.90	-
R01A	250	8	1	MD	129.49	-

R01A	250	8	2	MD	137.01	-
R01A	250	8	3	MD	130.71	-
R01A	250	15	1	MD	105.21	-
R01A	250	15	2	MD	98.36	-
R01A	250	15	3	MD	95.52	-
R01A	250	22	1	MD	74.13	-
R01A	250	22	2	MD	83.18	-
R01A	250	22	3	MD	89.76	-
R01A	250	28	1	MD	61.08	-
R01A	250	28	2	MD	68.20	-
R01A	250	28	3	MD	69.54	-
L6S	250	0	1	MD	159.65	-
L6S	250	0	2	MD	168.15	-
L6S	250	0	3	MD	163.37	-
L6S	250	1	1	MD	156.54	-
L6S	250	1	2	MD	159.62	-
L6S	250	1	3	MD	160.00	-
L6S	250	3	1	MD	163.61	-
L6S	250	3	2	MD	173.37	-
L6S	250	3	3	MD	166.81	-
L6S	250	8	1	MD	139.85	-
L6S	250	8	2	MD	138.61	-
L6S	250	8	3	MD	133.23	-
L6S	250	15	1	MD	115.70	-
L6S	250	15	2	MD	118.12	-
L6S	250	15	3	MD	126.82	-
L6S	250	22	1	MD	90.02	-
L6S	250	22	2	MD	95.19	-
L6S	250	22	3	MD	124.73	-
L6S	250	28	1	MD	87.46	-
L6S	250	28	2	MD	86.45	-
L6S	250	28	3	MD	108.43	-
R01A	500	0	1	MD	337.79	-
R01A	500	0	2	MD	333.47	-
R01A	500	0	3	MD	346.72	-
R01A	500	1	1	MD	271.45	-
R01A	500	1	2	MD	261.45	-
R01A	500	1	3	MD	313.73	-
R01A	500	3	1	MD	262.28	-
R01A	500	3	2	MD	246.38	-
R01A	500	3	3	MD	257.68	-
R01A	500	8	1	MD	234.89	-
R01A	500	8	2	MD	223.71	-
R01A	500	8	3	MD	216.20	-
R01A	500	15	1	MD	202.74	-
R01A	500	15	2	MD	203.39	-
R01A	500	15	3	MD	205.02	-
R01A	500	22	1	MD	153.91	-
R01A	500	22	2	MD	151.19	-
R01A	500	22	3	MD	162.77	-
R01A	500	28	1	MD	140.44	-
R01A	500	28	2	MD	141.03	-
R01A	500	28	3	MD	142.05	-
L6S	500	0	1	MD	340.23	-
L6S	500	0	2	MD	360.71	-

L6S	500	0	3	MD	320.03	-
L6S	500	1	1	MD	314.88	-
L6S	500	1	2	MD	308.21	-
L6S	500	1	3	MD	331.95	-
L6S	500	3	1	MD	326.46	-
L6S	500	3	2	MD	319.17	-
L6S	500	3	3	MD	357.56	-
L6S	500	8	1	MD	305.73	-
L6S	500	8	2	MD	313.89	-
L6S	500	8	3	MD	282.39	-
L6S	500	15	1	MD	277.01	-
L6S	500	15	2	MD	283.62	-
L6S	500	15	3	MD	263.33	-
L6S	500	22	1	MD	237.54	-
L6S	500	22	2	MD	254.00	-
L6S	500	22	3	MD	259.47	-
L6S	500	28	1	MD	209.93	-
L6S	500	28	2	MD	192.85	-
L6S	500	28	3	MD	199.40	-
R01A	50	0	1	PD+C	44.95	0.00
R01A	50	0	2	PD+C	47.13	0.00
R01A	50	0	3	PD+C	40.83	0.00
L6S	50	0	1	PD+C	36.13	0.00
L6S	50	0	2	PD+C	36.81	0.00
L6S	50	0	3	PD+C	35.51	0.00
R01A	50	1	1	PD	39.45	0.37
R01A	50	1	2	PD	35.06	0.27
R01A	50	1	3	PD	37.26	0.30
R01A	50	3	1	PD	29.93	1.05
R01A	50	3	2	PD	31.12	0.82
R01A	50	3	3	PD	30.07	0.98
R01A	50	8	1	PD	24.88	1.25
R01A	50	8	2	PD	30.36	3.79
R01A	50	8	3	PD	25.68	1.27
R01A	50	15	1	PD	21.61	0.86
R01A	50	15	2	PD	21.41	2.66
R01A	50	15	3	PD	21.77	1.53
R01A	50	22	1	PD	21.26	0.75
R01A	50	22	2	PD	19.23	0.93
R01A	50	22	3	PD	20.58	1.23
R01A	50	28	1	PD	15.50	0.68
R01A	50	28	2	PD	16.60	0.70
R01A	50	28	3	PD	15.10	1.07
L6S	50	1	1	PD	30.17	0.85
L6S	50	1	2	PD	31.60	0.74
L6S	50	1	3	PD	33.05	0.31
L6S	50	3	1	PD	29.31	0.65
L6S	50	3	2	PD	32.18	1.17
L6S	50	3	3	PD	30.34	0.86
L6S	50	8	1	PD	28.28	1.08
L6S	50	8	2	PD	28.32	1.82
L6S	50	8	3	PD	26.90	2.25
L6S	50	15	1	PD	25.21	1.46
L6S	50	15	2	PD	22.55	3.45
L6S	50	15	3	PD	21.75	1.71

L6S	50	22	1	PD	18.41	2.19
L6S	50	22	2	PD	18.96	7.32
L6S	50	22	3	PD	21.61	1.66
L6S	50	28	1	PD	21.39	1.36
L6S	50	28	2	PD	19.98	1.17
L6S	50	28	3	PD	21.14	1.13
R01A	50	1	1	C	39.19	0.00
R01A	50	1	2	C	40.95	0.00
R01A	50	1	3	C	42.49	0.00
R01A	50	3	1	C	39.31	0.24
R01A	50	3	2	C	36.05	0.22
R01A	50	3	3	C	39.67	0.40
R01A	50	8	1	C	36.60	0.72
R01A	50	8	2	C	36.05	0.38
R01A	50	8	3	C	36.94	0.33
R01A	50	15	1	C	35.60	0.82
R01A	50	15	2	C	33.46	0.80
R01A	50	15	3	C	34.32	0.58
R01A	50	22	1	C	32.77	0.74
R01A	50	22	2	C	32.51	0.24
R01A	50	22	3	C	31.19	0.63
R01A	50	28	1	C	29.34	0.37
R01A	50	28	2	C	31.01	0.50
R01A	50	28	3	C	29.87	0.70
L6S	50	1	1	C	35.19	0.00
L6S	50	1	2	C	34.50	0.00
L6S	50	1	3	C	36.36	0.00
L6S	50	3	1	C	35.89	0.16
L6S	50	3	2	C	33.27	0.06
L6S	50	3	3	C	35.57	0.13
L6S	50	8	1	C	33.91	0.22
L6S	50	8	2	C	32.39	0.86
L6S	50	8	3	C	30.41	0.14
L6S	50	15	1	C	31.84	0.11
L6S	50	15	2	C	31.77	0.32
L6S	50	15	3	C	30.83	0.34
L6S	50	22	1	C	28.37	0.36
L6S	50	22	2	C	25.43	0.52
L6S	50	22	3	C	28.88	0.41
L6S	50	28	1	C	24.64	0.50
L6S	50	28	2	C	29.25	1.12
L6S	50	28	3	C	27.37	0.66

SI-2 Quality criteria and pretests

Soil sampling, preparation and pre-incubation

Soils were sampled by the provider and conditioned to meet the requirements of OECD guide 307. Soil sampling was carried out by Fraunhofer IME (Schmallenberg, Germany) for the "RefeSol 01-A" soil and by LUFA (Speyer, Germany) for the clay soil "LUFA 6S". Soil sampling during or immediately after long periods of drought (> 30 days), frost or flooding was avoided. Soil was prepared (removal of vegetation, larger soil organisms and stones, and sieving through a 2-mm sieve) within one week after sampling. Soil was stored in at 4°C under aerobic conditions for less than 1 month until used for degradation experiments. Before the main experiment was conducted moisture of both soils was adjusted to 40% of maximum water holding capacity to ensure optimal microbial conditions. The moisture-adjusted soils were pre-incubated in the dark at 20°C under aerobic conditions for 1 week to re-establish equilibrium of microbial metabolism following the change from storage conditions to incubation conditions.

Confirmation of the nominal concentration of the AFB1 fortification standard

It was verified that the measured concentration of the stock solution prepared by dissolving 10 mg of crystalline AFB1 in 20 mL of MeCN (see Methods) corresponds to the nominal concentration of 500 mg L^{-1}. For this purpose, 3 aliquots were taken and diluted to a nominal concentration of 5 µg L^{-1} and measured by HPLC-FLD and quantified via external calibration (see Methods). Concentrations of 4.95, 5.14 and 5.10 were determined. The obtained concentrations were checked for significant differences from the nominal concentration of 5 by one-sample t.test. The obtained test statistic confirms that there is no significant difference from the nominal concentration ($t(2) = 1.095$, $p=0.388$).

Glass Adsorption test

It was checked whether the dissipation of AFB1 in the abiotic control(C) over time was at least partly due to adsorption of AFB1 on the glass material. For this purpose, empty glass jars were fortified as for the abiotic degradation samples. Immediately after spiking and evaporation step (t0) and after 8 days (t8) of incubation, the fortified jars were subjected to the same analytical procedure as the soil samples and the concentration was determined via HPLC-FLD using external calibration. Spike recoveries were 94.8 ± 10.4 and 94.2 ± 3.50 for t0 and t8, respectively. No significant decrease in extractable concentration over time was observed ($t(2.4) = 0.1$, $p = 0.928$).

Homogeneity of spike distribution

Table 2. The homogeneous distribution of AFB1 in the sterile (PD+C) and nonsterile (MD) soils fortified with 0.5. 5, 50, 250 and 500 µg kg^{-1} AFB1 was checked by mean spike recoveries and relative standard deviation of spike revovery (in brackets) at day 0 in triplicate.

Soil	Type	AFB1 level (µg kg^{-1})	%-Recovery
R01A	MD	0,5	67(6)
		5	90(4)
		50	71(1)
		250	66(3)
		500	68(2)
L6S	MD	0,5	64(14)
		5	65(3)
		50	66(1)
		250	65(3)
		500	68(6)
R01A	PD+C	50	89(7)
L6S	PD+C	50	72(2)

SI-3 Chromatographic data

LC-HRMS chromatograms and spectra

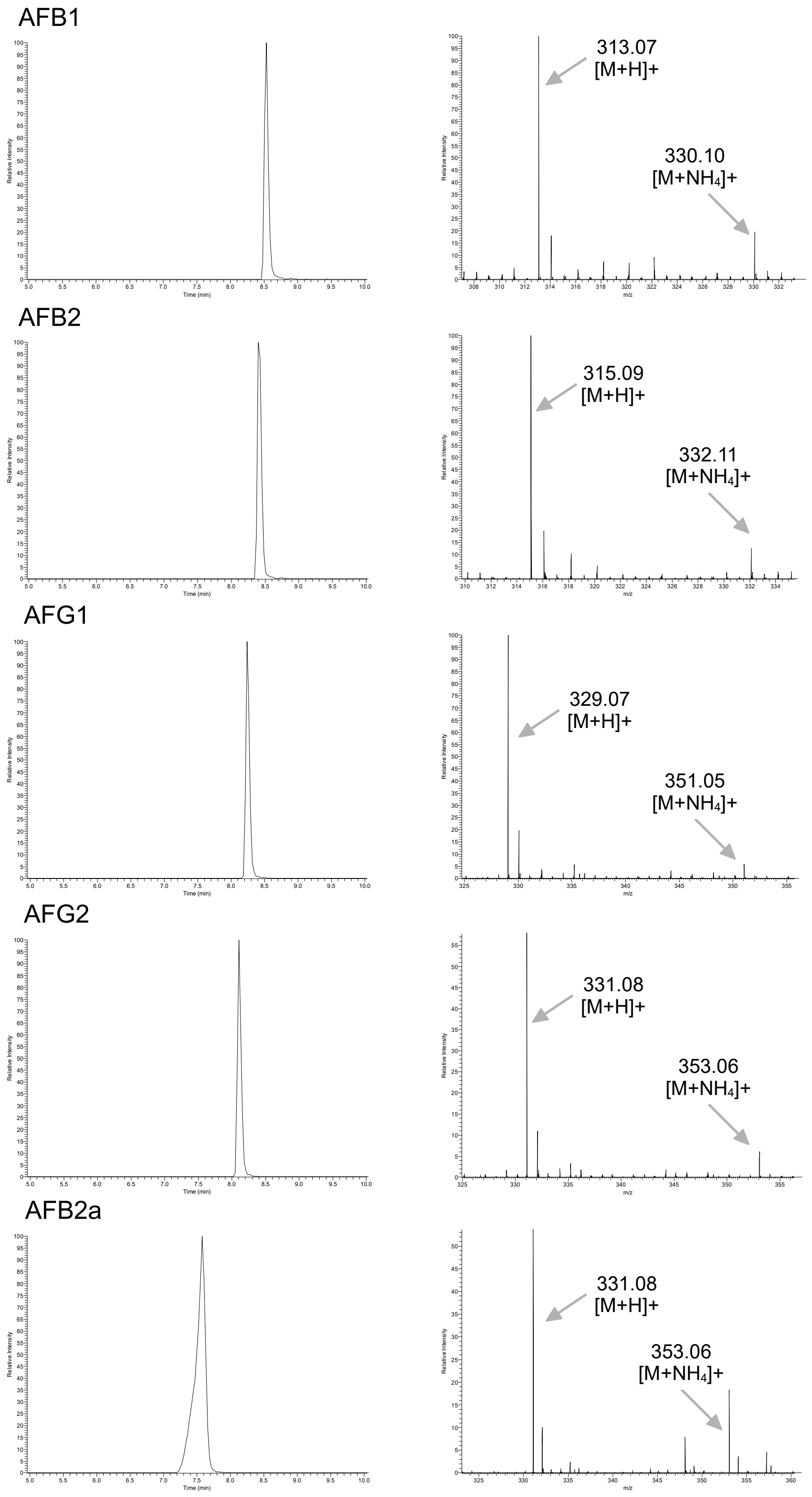

Figure 1. Chromatograms (left) and mass spectra (right) gained from LC-HRMS measurements of AFB1, AFB2, AFG1, AFG2 and AFB2a standards (5 µg L^{-1}).

HPLC-FLD chromatograms

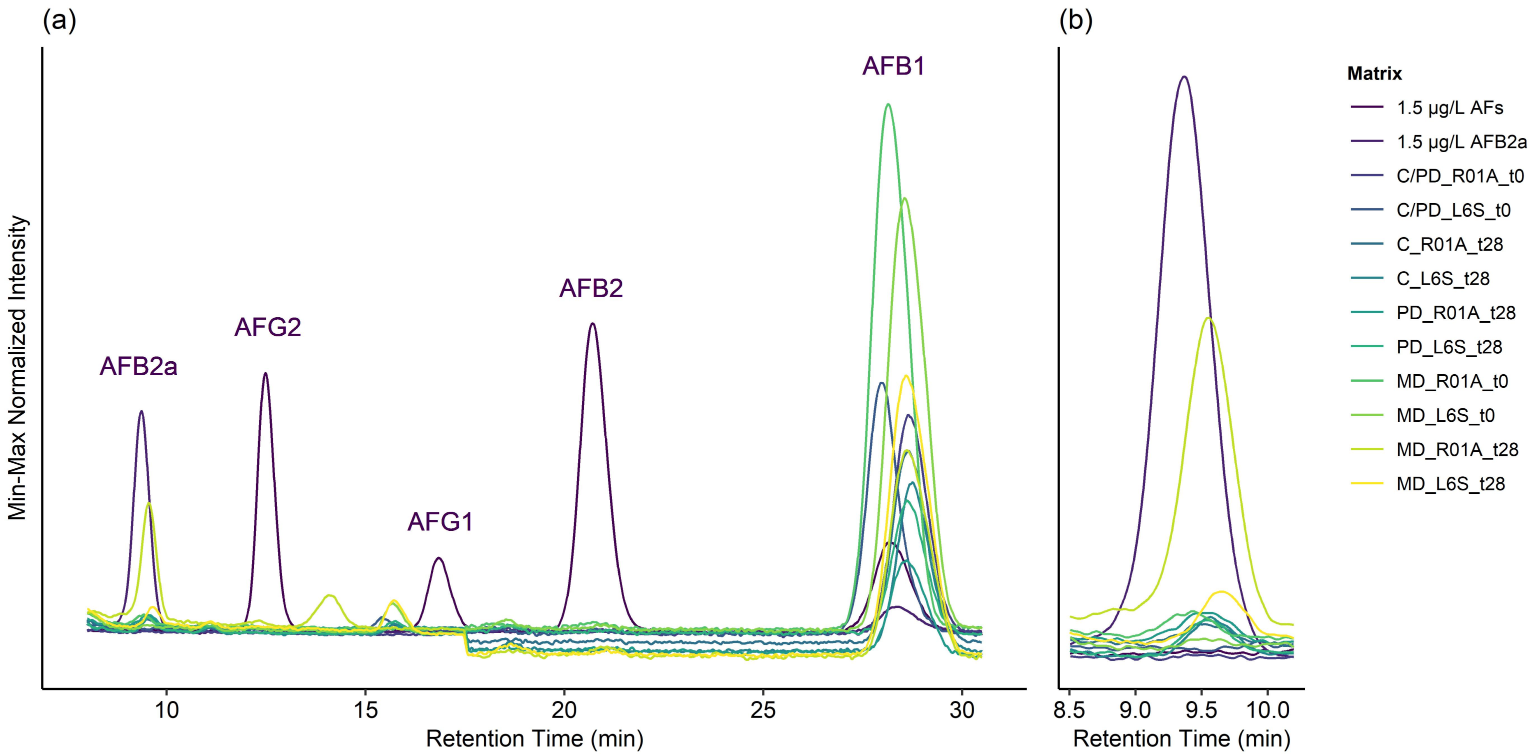

Figure 2. Chromatograms gained from HPLC-FLD measurements showing all investigated AFs i.e. AFB1, AFB2, AFG1, AFG2 and AFB2a Peaks (a) and a close up for AFB2a (b) in standards and extracts from day 0 and day 28. Extracts were obtained from sterile (PD+C) and nonsterile (MD) incubated soils fortified with 50 µg kg^{-1} AFB1.

SI-4 Statistical analyses

Statistical tests for the effect of incubation conditions on AFB1 dissipation

Table 3. ANOVA model summary: Effect of degradation type (MD, PD, C) and soil (sandy loam, clay) on AFB1 dissipation in terms of normalized AFB1 concentration at the end of 28-days incubation.

Predictor	DF_{effect}	DF_{error}	F	p
Degradation Type	2	12	72.15	<0.001
Soil	1	12	70.999	<0.001
Degradation Type:Soil	2	12	5.787	0.017

Table 4. Post-hoc test: Effect of the degradation type (MD, PD, C) for the individual soils (sandy loam, clay) on AFB1 dissipation in terms of normalized AFB1 concentration at the end of 28-days incubation.

Soil	DF_{effect}	DF_{error}	F	p
L6S	2	12	18.9	<0.001
R01A	2	12	59.1	<0.001

Table 5. Post-hoc test: Effect of the soil type (sandy loam, clay) for the individual degradation types (MD, PD, C) on AFB1 dissipation in terms of normalized AFB1 concentration at the end of 28-days incubation.

Degradation type	DF_{effect}	DF_{error}	F	p
C	1	12	4.7	0.051
MD	1	12	31.7	<0.001
PD	1	12	46.2	<0.001

Statistical tests for the effect of AFB1 initial concentration AFB1 dissipation due to microbial degradation

Table 6. Multiple regression summary: Effect of initial AFB1 level (0.5, 5, 50, 250, 500 µg kg^{-1}) and soil type (sandy loam, clay) on AFB1 dissipation in terms of normalized AFB1 concentration at the end of 28-days incubation due to microbial degradation.

Predictor	Estimate	t	p
(Intercept)	0.64 ± 0.02	39.2	<0.001
Initial AFB1 level	$(-1.41 \pm 0.65) \times 10^{-4}$	-2.2	0.040
Soil	-0.28 ± 0.02	-12.0	<0.001
Initial AFB1 level:Soil	$(2.61 \pm 0.92) \times 10^{-4}$	2.8	0.009

Table 7. Post-hoc test: Effect of the initial AFB1 level (0.5, 5, 50, 250, 500 µg kg^{-1}) for the individual soils (sandy loam, clay) on AFB1 dissipation in terms of normalized AFB1 concentration at the end of 28-days incubation.

Soil	DF_{effect}	DF_{error}	F	p
L6S	1	26	4.7	0.04
R01A	1	26	3.5	0.074

Table 8. Post-hoc test: Effect of soil type (sandy loam, clay) for the individual initial AFB1 levels (0.5, 5, 50, 250, 500 μkg^{-1}) on AFB1 dissipation in terms of normalized AFB1 concentration at the end of 28-days incubation.

AFB1 level ($\mu g\ kg^{-1}$)	DF_{effect}	DF_{error}	F	p
0.5	1	26	72.6	<0.001
5	1	26	60.9	<0.001
50	1	26	21.5	<0.001
250	1	26	19.1	<0.001
500	1	26	19.3	<0.001
